# Learning from the Two Case Reports of the Enriched Motor Intervention for the Development of Executive Functions in Children with Autism Spectrum Disorders

**DOI:** 10.3390/healthcare14121613

**Published:** 2026-06-08

**Authors:** Gabriele Gullo, Ambra Gentile, Chiara Rosaria Pace, Marianna Alesi

**Affiliations:** 1 Department of Psychology, Human Movement and Educational Sciences, University of Palermo, 90128 Palermo, Italy; gabriele.gullo01@unipa.it (G.G.); ambra.gentile01@unipa.it (A.G.); 2 Independent Researcher, 90100 Palermo, Italy; chiarapace13@gmail.com

**Keywords:** autism spectrum disorder, working memory, physical activities intervention program, executive functions

## Abstract

Background/Objectives: This study evaluates the feasibility and preliminary effects of a short version of the Enriched Motor Program (EMP), an intervention combining aerobic and cognitive exercises, adapted to support the development of executive functioning in children with autism spectrum disorder (ASD). Executive functions are high-order cognitive abilities necessary to manage everyday social and adaptive tasks. Methods: The sample included two children with ASD (boys) with an average chronological age of eight years. The intervention was delivered once per week, over 12 weeks, and children’s executive functions were assessed before and after the intervention. Working memory was assessed using the Backward Word Span and the Mr. Cucumber tests; inhibitory control was measured via the Circle Drawing Task and the Day/Night Stroop. Results: The data for both children were analyzed using the Reliable Change Index (RCI), which indicated a reliable change in visuo-spatial working memory for the child with level 2 ASD. Conclusions: These findings provide encouraging preliminary results concerning the feasibility of a short version of the EMP. Enriched motor programs could be considered as suitable activities complementary to the main clinical therapy.

## 1. Introduction

Following the DSM-5, Autism Spectrum Disorder (ASD) is a neurodevelopmental disorder with characteristics falling in two sub-domains: persistent deficits in social communication and social interaction across multiple contexts and restricted, repetitive patterns of behavior, interests, or activities. The diagnosis of the ASD condition is granted if a person shows symptoms in all three areas of the first sub-domain (deficits in socio-emotional reciprocity; deficits in non-verbal communicative behaviors; deficits in developing, maintaining and understanding relationships) and at least two of the second sub-domain (stereotyped or repetitive motor movements, use of objects or speech; adherence to routines, or ritualized patterns of verbal or non-verbal behavior; highly restricted fixated interests; hyper- or hypo-reactivity to sensory input or unusual interest in sensory aspects) [1]. Some typical characteristics, such as rigidity of behavior, stereotypes, preference for routines, resistance to change, impulsiveness, lack of shared attention, and pretend play, could be explained by damage to Executive Functions (EFs). Following Diamond’s [2] theoretical model, the three core EFs are working memory, inhibition, and cognitive flexibility. Working memory allows for retaining, manipulating, and updating information; inhibition allows for suppressing inappropriate stimuli; cognitive flexibility allows for switching between mental sets or perspectives to process information. These basic EFs cannot be considered separately in their actions, but act with areas of overlap in their functioning to flow into higher order processes such as reasoning, problem solving and planning. For example, inhibitory control improves the functioning of working memory by allowing it to work only on relevant information and preventing the intrusion of irrelevant or distracting stimuli. On the other hand, updating information allows for better inhibitory control to block information that has gradually turned out to be unnecessary. In turn, working memory and inhibitory control form the basis for cognitive flexibility, that is, the ability to move flexibly between rules and mental sets, monitoring the information active in memory) [3,4,5,6]. Although the association between ASD and deficits in EFs is well recognized, results are conflicting regarding which EFs are most compromised. In fact, some authors argue that planning and inhibition are more impaired [7], while others believe that working memory [8] or flexibility are more affected [9,10].

Moreover, impairment in EFs appears to be a factor predicting the level of condition severity. For example, deterioration in everyday EFs in two-year-olds is associated with more severe problems in emotional and behavioral domains [11], and deterioration in three-year-olds is a predictive factor of an increase in externalizing problems at five years old in children with ASD [12].

There is agreement on the exponential growth in the prevalence of ASD diagnosis in recent times, due to various factors such as an increase in environmental and epigenetic risk factors, as well as the development of more sensitive and reliable diagnostic tests [13]. The worldwide rates show an average of 100 per 10,000 with a range from 1.1 per 10,000 to 436.0 per 10,000 [14]. Recently, Scattoni and colleagues [13] rated the prevalence of children with ASD aged 7–9 years in urban and rural areas of Northern, Central, and Southern Italy and estimated an approximate rate of 1 in 77 children with a male-to-female prevalence of 4.4:1.

This recent continuous growth emphasizes the need for intervention plans tailored to children with ASD needs to enhance potential skills in order to improve their adaptation to daily life and decrease social and school disparities [15]. Specifically, with regard to the strengthening of EFs, recent studies demonstrated the beneficial effects of physical activity (PA) in enhancing EFs in children with ASD [16,17]. Villodres et al. [18] carried out a systematic review to summarize research examining the effects of PA interventions on executive function (EF) in 6–12-year-old children with ASD and attending primary school. They found beneficial effects on EF from innovative programs such as exergames or traditional PA programs such as basketball, table tennis, martial arts, and Chinese mind–body exercise, adequately integrated with ad hoc instructions and prompts [18]. The PA category can be considered in a broad sense, ranging from aerobic or circuit exercises to individual or team sports. As an innovative intervention program, video games have been revealed to be efficacious in improving EFs in children with ASD. For example, an intervention using the Xbox Kinect with the tennis game training skills as reaction time, balance, speed, and strength, associated with a warm demanding active stretching movements for the upper limbs, jumping, and hopping and cool down with yoga poses, static stretches, and deep breathing was more effective compared to the SPARK (Sports, Play and Active Recreation for Kids), a traditional motor training intervention [19]. As a traditional motor intervention, Arslan and coll. investigated the effectiveness of a 12-week circuit exercise program by comparing motor and cognitive performances in 14 boys with ASD and 14 boys with typical development. Results showed how typically developing children participating in the exercise program of three 60 min sessions per week for 12 weeks improved cognitive skills, such as reaction time, motor skills, running speed and agility, balance, handgrip strength, and flexibility. The ASD group was additionally trained with skill instruction and controlling prompts indoors, and improvement was found in auditory reaction time, balance, standing long jump and handgrip strength [20]. Phung and Goldberg [21] showed the positive effects of a mixed martial arts (MMA) 26-class program over a 13-week program tailored to school-aged children with ASD at increasing EFs, such as behavioral inhibition, working memory, and cognitive flexibility. The authors underlined the greater effectiveness of martial arts with a traditional approach in improving EFs compared to modern ones because the former also train self-discipline with behavioral, cognitive and emotional control [21]. Even a 12-week mini-basketball training program (MBTP), delivered to preschool children with ASD, increased EFs in an enjoyable and safe way. Improvement in EFs is stimulated by progressively increasing the cognitive load of exercises, such as working memory, to update and manipulate spatial information for the position of the players on the field and the ball movement, and neurocognitive demands to perform motor-cognitive tasks, associated with opportunities of social interaction and communication [22]. On the other hand, also a 12-week table tennis intervention with two 70 min sessions per week, consisting of 5 min of warm-up, 20 min of motor skill exercises, 20 min of table tennis and 5 min of cool down was useful to increase scores on correct response and conceptual level and decrease perseveration in children with ASD [23].

Widely recognized are the benefits of evidence-based programs with structured activities delivered in the aquatic environment to improve not only motor skills, but also social relationships, language development, self-confidence and mastery motivation [24,25]. The use of evidence-based motor programs, aimed at developing EFs, to be delivered in the school or extra-school context is also spreading for children with ASD. This is the case of the Enriched Motor Program, EMP (original title: PMA. Programma Motorio Arricchito [26]), which is a teacher-led program built to enhance EFs, such as inhibitory control, working memory, cognitive flexibility, and planning, in typically developing preschoolers, through cognitively challenging movement tasks, following Diamond’s [27] and Diamond and Ling’s [28] recommendations.

EMP is composed of 60 min sessions lasting 30 sessions that consist of gross-motor tasks (such as throwing, catching, bouncing, kicking, pulling, and pushing) and fine-motor tasks (such as using crayons and scissors, copying shapes and letters, blinking, buttoning clothes, etc.). Each session is enriched by a cognitive task aimed at stimulating EFs, such as Animal or Fruit Stroop tasks, body parts or words span tasks, a maze, etc. In our previous study [29], we hypothesized the efficacy of EMP to improve motor skills and EFs in children with ASD, based on previous findings showing how the program resulted in physiologically and cognitively beneficial outcomes for typically developing children [26,30]. So, the EMP was adapted and reduced to twelve sessions to be suitable for the cognitive and physical needs of children with ASD.

Although the potential benefits of motor programs on both the physical and mental health of children with ASD are theoretically recognized, research in this area remains limited and is characterized by conflicting results. Also, to the best of our knowledge, this is the first attempt to use cognitive enrichment with tailored stimuli created specifically from the neuropsychological literature, while most of the previous studies in the literature take advantage of the cognitive demands of different sports.

## 2. Materials and Methods

### 2.1. Study Design

The present feasibility pilot study was structured into three phases:

- Pre-intervention assessment (T_0_). At baseline, participants underwent a comprehensive evaluation of executive functions using four standardized measures assessing children’s working memory (WM) (two measures: one for visuospatial WM and one for verbal WM) and inhibitory control (two measures: one for verbal inhibition and one for behavioral inhibition).
- Intervention phase. Participants took part in the short form of the Executive–Motor Program (EMP), consisting of 12 structured activities. The intervention was initially planned to be delivered three times per week over a four-week period; however, modifications were made to accommodate participants’ schedules, as the children were also engaged in group-based social skills training and personal activities; therefore, the intervention was delivered once per week across a 12-week period.
- Post-intervention assessment (T_1_). Following the intervention, the same four standardized measures were re-administered to explore post-intervention changes in executive functions at the individual level.

### 2.2. Enriched Motor Program [EMP]

#### 2.2.1. Intervention Design

The intervention consisted of a short form of the Enriched Motor Program (EMP), comprising twelve units of cognitively enriched motor activities (for a detailed description of all activities, see Appendix A). Each unit lasted 45 min and was delivered once per session. Each session was structured into two consecutive phases: a 15 min warm-up phase and a 30 min cognitively enriched motor activity phase.

The warm-up phase included dynamic stretching exercises (e.g., shoulder rolls, head circles, lunges) combined with aerobic exercises (e.g., running and jumping jacks). This phase is aimed at preparing participants for the subsequent motor-cognitive activities.

The cognitively enriched motor activity phase included one structured activity per session, each specifically designed to target a single executive function (EF). Across the twelve units, five activities targeted working memory, five targeted inhibitory control, one focused on cognitive shifting, and one addressed planning abilities. Each activity was repeated across sessions with systematic variations in task rules, motor sequences, or cognitive demands to maintain engagement and provide progressive executive function stimulation. For example, in a working memory activity, the experimenter verbally named a sequence of body parts that the child was required to touch in the same order, with the sequence length progressively increasing from two to four items; in a variation of this task, the child was instructed to reproduce the sequence in the reverse order.

#### 2.2.2. Application

To ensure maximal adherence to the study procedures and to minimize potential discomfort for the children, both the assessment sessions and the intervention activities were conducted within the facilities of the psychosocial center.

All activities were carried out by two researchers who had received specific training on EMP activities, in collaboration with the children’s therapists, who were present throughout the sessions and were responsible for supervising behavioral management (e.g., intervening in case of aggressive behaviors).

### 2.3. Participants

The study included two children diagnosed with ASD according to DSM-5 criteria [1]. The sample consisted of two males aged 7 and 9 years old. The participants were recruited from a private psycho-educational center in Palermo, specialized in neurodevelopmental disorder treatment.

Detailed clinical diagnosis was not directly administered by the research team but was retrieved from the public health service diagnosis and certification. The clinical team did not share data such as children’s functional profiles due to privacy restrictions. Eligibility was determined by the clinical team of the psycho-educational center based on existing clinical records provided by the public health service. These professionals identified participants in accordance with the following predefined inclusion criteria: (1) to have received an ASD diagnosis; (2) to not have co-occurring diagnosis of ADHD nor Disruptive, Impulse-Control, and Conduct Disorders; (3) to be between six and eight years old or have an equivalent mental age; (4) to master language skills such as early forms of understanding, listening, and expressing, i.e., pointing to body parts, following instructions, listening and paying attention, the use of names, sentence language, asking questions; and (5) to master motor skills including the fundamental motor skills (FMS) of standing alone and walking, crawling, running, walking up/down stairs, jumping, throwing, and catching.

Parents gave their consent to participate in the study. The consent form described the aims, procedures, and methodology of the study. Parents were also informed about the procedures for data management, including storage, protection, and anonymization of personal information. It was clearly explained that all data would be treated confidentially and reported in a non-identifiable form. In addition, parents were asked to provide specific consent for the publication of the study results in peer-reviewed scientific journals, with the assurance that no personal or sensitive data that would allow participant identification would be disclosed. Parents were not asked for consent to take photos.

Given the exploratory nature of the study, participants are described individually, in line with methodological recommendations for intervention studies involving single-case research [31].

#### 2.3.1. Participant 1

Participant 1 was a 7-year-old male child who had been certified by a public institution (ASP—Azienda Sanitaria Provinciale). The documentation at our disposal reported a diagnosis of ASD, with adequate cognitive development (general IQ = 107). The child did not present any evidence of neurological impairments or speech and language development disorders. Functional verbal language was present at baseline.

#### 2.3.2. Participant 2

Participant 2 was a 9-year-old male child who had been certified by a private clinical practitioner. The documentation at our disposal reported a diagnosis of Level 2 ASD (i.e., requiring substantial support), with adequate cognitive development (global IQ not reported). The child presented difficulties in visual attention and in maintaining eye contact. The child did not present any evidence of neurological impairments or speech and language development disorders. Functional verbal language was present at baseline.

### 2.4. Measures

#### 2.4.1. Mr. Cucumber Test [32]

Children’s visuospatial working memory was assessed using the Mr. Cucumber Test, where they are asked to view a cartoon alien figure with colored stickers placed on various body parts. The task consists of remembering and then recalling the spatial locations of these stickers by indicating the exact positions on a blank outline of the same alien. The test consists of eight levels, each one corresponding to the number of sticker locations to be recalled (1–8). At each level, children need to complete three unique trials, for a total of 24 trials. For Levels 1–5, each stimulus is presented for 5 s, while from Level 6 onward, the exposure time increases by one second per level (i.e., 6 s at Level 6, 7 s at Level 7, and 8 s at Level 8), providing additional time as memory demands increase. The reported Cronbach’s alpha for this test is α = 0.65.

#### 2.4.2. Backward Word Span [33]

Backward Word Span was chosen for assessing verbal working memory. The task consists of a series of lists of semantically unrelated two- or three-syllable words. Children had to recall this list in the reverse order. A practice trial (corrected if necessary) is provided to show children how the word lists should be repeated backward, followed by the test trials. Each level consists of three items (i.e., three series of words), starting with two words and increasing by one at each level, until the participant fails to recall at least two of the three items of the same length, or until the maximum length of seven words is reached. The score is the number of words at the highest consecutive level that the participant repeated correctly in reverse order in at least two lists, plus one-third of a point for each correct list beyond that length. Cronbach’s alpha reported by the Authors for this test is α = 0.79.

#### 2.4.3. Circle Drawing Task

Circle Drawing task from the EF-PS 2-6 Battery [34] was used to assess children’s motor inhibitory control. The task requires tracing a circle drawn on a white sheet of paper with a finger. The execution speed should be adapted to the examiner’s requests. In the first execution, children are not informed about the speed of execution, while in the second execution, they are required to modulate their motor response, doing the task as slowly as possible. The score corresponds to the difference between the second and the first execution’s time, converted into seconds. The manual reported Cronbach’s alpha of 0.57.

#### 2.4.4. Day and Night Stroop Task

Day and Night Stroop Task from the EF-PS 2-6 Battery [34] was used to assess inhibitory control. In this task, children are presented with a card of the sun or of the moon. The child must inhibit the dominant response (e.g., saying “day” when presented with a card featuring the sun) in favor of the non-dominant response (saying “night” when a card with the sun is shown). This task requires both inhibitory control and working memory to recall the rule. However, the demands on working memory are modest and do not significantly affect performance [35]. The Cronbach’s alpha reported for this test is α = 0.86.

### 2.5. Data Analysis

The Reliable Change Index (RCI) was used to describe within-individual change between pre- and post-intervention assessments [36]. The RCI is calculated as a ratio in which the numerator represents the difference between two measurements (e.g., pre- and post-intervention scores), while the denominator represents an estimate of the standard error associated with that difference [36]. In its conventional formulation, RCI values are expressed on a standardized metric analogous to a *z* score, with a mean of 0 and a standard deviation of 1; values such as ±1.96 are commonly reported in the literature as reference benchmarks for changes exceeding expected measurement variability [37].

In the present study, RCIs were used solely as descriptive indicators of the magnitude and direction of within-individual change to support the interpretation of case-level variation in this exploratory feasibility pilot. Given the single-case design, no inferential statistical conclusions were drawn, and RCI results were considered hypothesis-generating information intended to inform future replication in larger and methodologically stronger studies [38,39].

## 3. Results

Pre-intervention and post-intervention measures of executive functions for each participant, with the corresponding RCIs, are reported in Table 1.

Participant 1’s RCIs fell within the nonsignificant range across all measured variables, indicating no reliable change in performance across the assessed domains of executive functioning.

Participant 2 showed a reliable change only in the Mr. Cucumber task, with an RCI of 2.88, demonstrating a reliable change in performance in the visuo-spatial working memory domain, whereas the other RCIs fell within the ±1.96 threshold, indicating stable performance across the assessment.

## 4. Discussion

RCI values for Participant 1 remained within the expected range across all assessed variables, indicating no notable within-individual change in performance across the executive functioning domains examined.

From a descriptive clinical perspective, the child showed good levels of compliance and cooperation with both experimenters throughout the assessment sessions and EMP activities. These observations were based on non-systematic clinical impressions. Occasional episodes of frustration were noted following more challenging tasks (e.g., difficulty catching the ball promptly or missing the skittles), during which the child complained or displayed brief signs of distress (e.g., growled while gritting his teeth). Such episodes lasted a few minutes and were effectively managed by the therapists, who supported the child in regulating his emotions, suggesting different strategies (e.g., squeezing a stress ball). No additional aggressive behavior was observed. Overall, the child remained engaged and motivated, particularly during activities involving running or gross motor coordination.

Participant 2 showed an increase in performance on the Mr. Cucumber task, with an RCI value of 2.88, indicating a within-individual change in visuo-spatial working memory.

From a descriptive clinical perspective, the child demonstrated good compliance and cooperation with both experimenters throughout the assessment sessions and EMP activities. No signs of distress, frustration, or aggressive behavior were observed, and the child remained consistently engaged and enthusiastic during participation. During heel-to-toe walking tasks, difficulties were noted in maintaining direction and discriminating between pathways. So, the spacing between the pathways was increased, after which task execution improved, and no further pathway confusion was observed. This adjustment reflects the adaptive and feasibility-oriented nature of the intervention rather than the outcome of the program.

To sum up, the only relevant result is about the RCI in participant 2, which reveals a reliable change in visuospatial working memory only. Most activities systematically relied on visual attention and provided dynamic visual cues as well as selective processing of visual information (e.g., identifying an animal from its head or some fruit from its core). These demands require children to focus on salient stimulus characteristics while filtering irrelevant information, which is commonly reported as challenging for children with ASD [40]. From a theoretical perspective, this aspect of the program should be further deepened, since previous research showed that visuomotor training is connected with reductions in repetitive behaviors [41], suggesting broader regulatory effects beyond motor performance alone. Moreover, visuospatial working memory is also closely linked to spatial perspective-taking [42], which is the ability to represent space from viewpoints other than one’s own [43].

No reliable changes were found in verbal working memory or in verbal and motor inhibitory control for both participants. However, the lack of reliable changes in verbal and motor inhibitory control could have been possibly influenced by the measurement tools used for the assessment. Regarding the verbal inhibitory control, it is worth noticing that both participants obtained the maximum scores at both pre- and post-intervention assessments, suggesting a potential ceiling effect that may have masked any possible changes [42]. Additionally, a plausible explanation for the lack of reliable changes in motor inhibitory control is that the Circle-Drawing Task requires children to continuously modulate drawing speed, engaging sustained and regulated motor control during an ongoing movement rather than the discrete suppression of a prepotent response. This fundamentally differs from classic “go/no-go” paradigms, which rely on discrete response inhibition [44]. On the other hand, EMP activities are more focused on motor response inhibition, more closely aligned with the demands of a “go/no-go” task, potentially explaining the absence of reliable changes. Motor learning in children with ASD is frequently context-bound, needing an explicit form of guidance [45]. Therefore, as EMP activities focus on binary “stop” and “go” responses rather than on the modulation of speed, enhancements in discrete motor inhibition may not have been detected by the circle drawing task.

## 5. Limitations and Future Directions

While the present findings provide preliminary insights for further investigations on the adaptation of the EMP to children with ASD, some methodological limitations should be noted.

Since this was a pilot feasibility study reporting the case of two children, it does not allow for the generalization of the results, nor does it permit firm conclusions regarding the efficacy of the EMP. Future research should consider a different research design (e.g., case–control studies) involving larger, well-powered samples and controlled designs (e.g., randomized design) to strengthen generalizability and examine the program’s effectiveness.

Furthermore, the lack of detailed information on each child’s developmental history and functional profile, due to privacy restrictions, limits the possibility of examining how individual factors (e.g., ASD core symptoms) may have influenced the observed changes.

Another important limitation concerns the frequency of intervention delivery. As already acknowledged in the Methods section, accommodations to the intervention delivery frequency were necessary due to the children’s and therapists’ busy schedules. Consequently, the EMP was administered only once per week, which may have limited the program’s potential benefits. According to a meta-analysis by Wu et al. [46], activities should be administered at least three times per week to enhance EFs effectively. Future studies should consider increasing session frequency to align with established recommendations for effective EF intervention.

## 6. Conclusions

In this exploratory feasibility pilot study, the EMP was found to be acceptable and manageable for both participating children, who engaged with the activities despite their challenging nature. Only small adjustments were made to the original version of the maze activity to make it more manageable for participant 2. The reliable change observed in visuospatial working memory in Participant 2 suggests a potential area of interest for further investigation.

Adopting a structured motor program which integrates ad hoc cognitive stimuli may be beneficial for children with ASD’s executive functioning. Specifically, given that the activities of the program are delivered as ludic and enjoyable tasks, similar programs might be suitable as complementary intervention programs to the main clinical therapy.

## Figures and Tables

**Table 1:** Executive Functioning results at pre-intervention (T_0_) and post-intervention (T_1_) assessment and RCIs.

Participants		MrCu ^1^	WBS ^2^	CDT ^3^	DNS ^4^
Participant 1	T_0_	3	2	0.51	16
	T_1_	2.99	2	0.56	16
	RCI	−0.01	0	0.19	0
Participant 2	T_0_	1	2	0.56	16
	T_1_	3	2.33	0.44	16
	RCI	2.88 *	0.61	−0.45	0

^1^ Mr. Cucumber test; ^2^ Words Backward Span; ^3^ Circle Drawing Task; ^4^ Day/Night Stroop. * Significant RCI’s value.

## Data Availability

Data sharing is not allowed due to legal restrictions and is available under formal request addressed to the Bioethical Committee of the University of Palermo.

## Abbreviations

The following abbreviations are used in this manuscript:

EMP	Enriched Motor Program
ASD	Autism Spectrum Disorder
RCI	Reliable Change Index
EF(s)	Executive Function(s)
EEF(s)	Everyday Executive Function(s)
PA	Physical Activity
SPARK	Sport, Play and Active Recreation for Kids
MMA	Mixed Martial Arts
MBTP	Mini-Basketball Training Program
PMA	Programma Motorio Arricchito
WM	Working Memory
ADHD	Attention-Deficit/Hyperactivity Disorder
FMS	Fundamental Motor Skills
IQ	Intelligence Quotient
EF-PS	Batteria per la valutazione delle Funzioni Esecutive in età Prescolare [Pre-Schoolers Executive Functions Assessment Battery]
MrCu	Mr. Cucumber Task
WBS	Words Backward Span
CDT	Circle Drawing Task
DNS	Day/Night Stroop

## Appendix A. EMP Activities (from Gullo et al. [29])

### Appendix A.1. Unit 1

Children run and pass the ball to one another, calling each other by name while avoiding collisions with their peers. After receiving a signal from the teacher, the pairs reassemble with different partners to continue the activity. Each child then throws the ball against the wall and is required to name as many animals (or, alternatively, colors or fruits) as possible in one minute.

#### Variation

Children form a circle and pass the ball to a casual partner. The one who passes the ball also chooses a different category (e.g., food, transportation, etc.) while the catcher must say as many words as possible in one minute.

### Appendix A.2. Unit 2

After a warm-up phase, children lie down on their backs silently to listen to their heartbeats for at least two minutes. Subsequently, children are required to perform specific movements, such as clapping hands or stomping feet, in accordance with the rhythm of the music.

#### Variation

Sessions with varying alternate rhythms and movements.

### Appendix A.3. Unit 3

The experimenter places two mats on the floor: one composed of cotton tufts, representing the snow, and one made with green stripes, representing the grass. Children must reach one of the mats using different forms of movement decided by the experimenter (crawling, walking, jumping, etc.) and touch one of the mats. The rule consists of touching the snow mat when the experimenter says the word “grass” and touching the grass mat when the experimenter says the word “snow”.

#### Variation

The experimenter indicates the color of the mats instead of naming them “snow” and “grass”.

### Appendix A.4. Unit 4

The experimenter first names sequences of body parts, and then the child must touch them following the same order, starting with two parts (e.g., eyes–mouth), increasing to three (e.g., nose–ear–mouth) and then four parts (e.g., mouth–nose–ear–eyes). Afterwards, the experimenter names sequences of body parts, but the child must touch them in reverse order. For instance, if the experimenter says “eye-mouth”, the child should touch “mouth-eye”.

### Appendix A.5. Unit 5

The experimenter names a sequence of animals, and the child must imitate the gait of the mentioned animals following the same order, starting with two animals (e.g., dog–frog), increasing to three (e.g., dog–snake–frog) and then four parts (e.g., frog–dog–kangaroo–cat). Afterwards, the experimenter names animals, but the child must imitate their gait in reverse order. For instance, if the experimenter says “dog-frog”, the child should imitate the gait of “frog-dog”.

### Appendix A.6. Unit 6

The experimenter places two pairs of pins on the ground, each showing Stroop images of fruits (i.e., images consisting of two fruits one in another). The experimenter asks children to focus on the smaller fruit, and the child runs around the pin with that fruit. The pairs are pineapple–pear and strawberry–lemon.

#### Variation

The experimenter asks to focus on the smaller fruit, and the child should try to take down the right pin with a bowling ball.

### Appendix A.7. Unit 7

The experimenter places two pairs of pins on the ground, each showing Stroop images of animals (i.e., images with the head of one animal and the body of another). The experimenter asks the child to focus on the head of the animal, and the child runs around the pin with the head chosen by the experimenter. The pairs are squirrel–snake and giraffe–zebra.

#### Variation

The experimenter asks the children to focus on an animal, and the child should try to take down the right pin with a bowling ball.

### Appendix A.8. Unit 8

The experimenter creates an obstacle course for children using available gym materials such as hoops in pairs of different colors, cones, mattresses, and baskets. When children reach the pairs of hoops, they should jump into the correct hoop according to the incongruent rule: If the experimenter says RED, the child should jump into the BLUE hoop; conversely, if the experimenter says BLUE, the child should jump into the RED hoop.

#### Appendix A.8.1. Variation 1

When children reach the pairs of hoops, they should jump into the correct hoop. The congruent rule is the following: if the experimenter says a color (e.g., red), the child should jump on the hoop with the same color.

#### Appendix A.8.2. Variation 2

When children reach the pairs of hoops, the experimenter will articulate words with the same initial letter, emphasizing the starting sound to give the impression that they are going to say the color of the hoop (e.g., for the red hoop, the experimenter could say “rrrr-rare”, “rrrr-rain”, etc.).

### Appendix A.9. Unit 9

The experimenter creates four zones with colored tapes (blue, yellow, green, and red) or, alternatively, hoops of different colors. Children are required to reach the zone of the color mentioned by the experimenter and perform an associated movement, following these indications: when the experimenter says “blue”, children have to make a circle; when the experimenter says “yellow”, children should line up in order of height; when the experimenter says “green”, children should spin around; when the experimenter says “red”, children should roll on the mat.

### Appendix A.10. Unit 10

The experimenter constructs a labyrinth using three colored tapes to delineate three distinct paths. Seven stuffed animals are placed at various locations within the maze, ensuring that one path contains a greater number of animals than the others. Children are required to select the path enabling them to collect the maximum number of animals possible. The child must free one animal at a time, adhering to the established rules while choosing the appropriate path.

#### Variation

The experimenter asks children to free two specific animals. Sometimes, the animals are on the same path, while sometimes they are not. Therefore, children must decide if it is possible to save both animals by choosing one path or not.

### Appendix A.11. Unit 11

The experimenter sets up a relay course with the available gym materials. Children have to follow the rules for completing the course, such as to pass under an obstacle, jump on one foot, and walk on a line. The experimenter manages two puppets (a frog and a dragon) to give dispositions to children to start running, but they are required to follow the instructions provided by the frog. Therefore, children must wait for the “GO” from the frog and stay still if the “GO” is given by the dragon.

### Appendix A.12. Unit 12

The experimenter distributes scissors and paper images. Each sheet contains two different drawings that are a star formed by small moons and a moon formed by small stars, a moon formed by small suns and a sun formed by small moons, a sun formed by small stars and a star formed by small suns. Other sheets contain the letter A formed by small Es and the letter E formed by small As, the letter O formed by small Us and the letter U formed by small Os. The experimenter requires the child to focus only on one of the two elements present (e.g., MOON), and the children have to cut out the image composed of the selected element (e.g., SUN formed by small MOONS).

#### Variation

The experimenter asks to focus on the two elements present (e.g., SUN), and children will draw the image composed of the selected element (e.g., MOON formed by small SUNS).
